# Clinical and Demographic Characteristics of Women with Intrauterine Adhesion in Abuja, Nigeria

**DOI:** 10.1155/2012/435475

**Published:** 2011-12-01

**Authors:** Efena R. Efetie, Augustine C. Umezulike, Ugochukwu V. Okafor

**Affiliations:** ^1^Department of Obstetrics and Gynaecology, National Hospital Abuja, Central District, PMB 425, Garki, 900001 Abuja, Nigeria; ^2^Department of Anaesthesia, National Hospital Abuja, Central District, PMB 425, Garki, 900001 Abuja, Nigeria

## Abstract

*Objective*. Infertility menstrual abnormalities continue to constitute a significant bulk of gynecological consultation in Africa. Both of these problems are sometimes traced to intrauterine adhesions which are preventable in the majority of cases. *Study Design*. A retrospective analysis of intrauterine adhesions at the National Hospital Abuja, Nigeria, was carried out, covering the period from 1st September 1999 to 1st September 2004. A total of 72 cases were analyzed. Statical analysis was done using *X* ^2^. *Results*. The incidence of intrauterine adhesions was 1.73% of new patients. Mean age ± SD was 29.97 ± 4.82 years. Patients who were Para 0 to 1 constituted 81.9% of the total. Intrauterine adhesions significantly (*P* < 0.02) occurred in nulliparae. The majority (68%) were educated only up to secondary level which was significant (*P* < 0.05). Menstrual abnormalities were present in 90.3%. The commonest predisposing factor identified was a history of dilatation and curettage or uterine evacuation. *Conclusion*. Intrauterine adhesions are associated with lower educational status and low parity. Increasing educational targets nationally, poverty alleviation, nationwide retraining in manual vacuum aspiration, and wider application of this technique are recommended.

## 1. Introduction

 Intrauterine adhesions (IUA) are also known by the term “uterine synechiae,” and Asherman's syndrome. They were first described by Fritsch in 1944, although Asherman increased awareness of the condition significantly [1, 2]. IUA presents clinically with menstrual abnormalities usually hypomenorrhoea, oligomenorrhoea, or secondary amenorrhea. Sometimes, it may present with cyclical abdominal pain or with recurrent abortions [3].

 The condition is caused usually by trauma to the endometrium, with infection occasionally contributing to the pathogenesis. Pregnant or recently pregnant uteri are more susceptible [1], and therefore IUA is more common in areas with high incidences of unsafe abortion [1]. A study in Nigeria implicated induced abortion in 23% of cases [4]. Other implicated factors include postpartum curettage, Caesarean section, myomectomy pelvic inflammatory disease, and repair of ruptured uteri [5]. 

 IUA can be diagnosed through various methods. Hysterosalpingography is the most commonly employed method. Other methods include hysteroscopy (with or without 3-dimensional features), ultrasonography, and magnetic imaging [6–9].

 Universally, the incidence of IUA is increasing mainly from curettage in induced, incomplete, and missed abortions, as well as in postpartum haemorrhage and also genital tuberculosis [10–13]. The manual vacuum aspiration (MVA) is a less hazardous procedure, with regard to complications such as IUA, compared to traditional dilatation and curettage [1, 14]. Training courses on this technique have been held in major cities in Nigeria, including Abuja, the Federal Capital Territory.

Bearing in mind the triad of poverty, ignorance, and disease, and the vicious cycle thus generated, this paper aims to highlight the clinical and demographic characteristics of women presenting with IUA at the new tertiary health facility in the Federal Capital Territory of Nigeria.

## 2. Materials and Methods

The case folders of patients with IUA who attended the gynaecological out-patients clinic of the National Hospital Abuja during a 5-year period between 1st September 1999 (the hospital inception date) and 1st September 2004 were retrieved from the medical records department. This hospital is the major specialist hospital serving the Federal Capital Territory of Nigeria.

 Diagnosis was made following a hysterosalpingogram (HSG) and a negative progesterone challenge test. In some cases, ultrasound examination aided the diagnosis but confirmation was usually with HSG. Strict anatomical criteria of IUA were used and so cased with diagnosis of cervical synechiae were excluded from the study.

 The age, parity, and educational status of the respondents were analyzed. Also the clinical presentation and any predisposing factors were analyzed. Level of significance was set at *P* < 0.05 (95% confidence interval). In 9 cases, there was more than 1 predisposing factor. In such cases, the most recent antecedent event was selected for the purpose of the study.

## 3. Results

During the period under study, there were 72 recorded cases of IUA out of a total of 4,165 new attendances at the gynaecological out-patients clinic. This gives a rate of 1.73%. All case-folders were retrieved and analysis was based on this figured as the denominator. The duration of symptoms ranged from 4 months to 7 years.

## 4. Age and Parity

The mean age of patients with IUA was 29.97 ± 4.82 years, with a range of 20–43 years. In terms of parity, 81.9% were of low parity (Para 0 or Para 1). These are show in Tables 1 and 2. IUA significantly occurred in nulliparae (*P* < 0.02, *X* ^1^ = 5.6, 1 df). 

## 5. Educational Status

The majority (65.3%) were educated up to secondary level, as opposed to those with postsecondary education, and this was statistically significant (*P* < 0.05, *X* ^2^ = 7.9, 1 df). Two patients had primary level education. This is shown in Table 3.

## 6. Clinical Presentation

Table 4 shows the clinical presentation of the patients. Menstrual abnormalities were present in 90.3% of cases.

## 7. Predisposing Factors

The predisposing factors are show in Table 5. The highest contribution factor was a history of dilatation and curettage or uterine evacuation, either for a spontaneous or induced abortion or postpartum haemorrhage. Next in ranking order was a history of myomectomy, Caesarean section, manual removal of retained placenta, and pelvic inflammatory disease. No predisposing factors were found in one case record.

## 8. Discussion

Comparison to state increase or decrease in prevalence is hindered by paucity of reports on the prevalence of IUA in the Federal Capital Territory of Nigeria. However, with reference to the study in Lagos, Nigeria [1], there is a lower prevalence of the condition in this region of the country. This may reflect the impact of the nationwide training in manual vacuum aspiration (MVA) techniques which began in 1989. 

 There were no patients in the under-20 age bracket while ages above 40 witnessed low numbers of patients. The majority (80.6%) were in the 25–35 age brackets, which is a reflection of the age pattern of reproductive activity. The mean age is similar to the study in Lagos, Nigeria [1].

 As in Lagos study, the majority (18.9%) had a low parity of Para 0-1, and in this study, IUA significantly occurred in nulliparae (*P* < 0.02), illustrating the low reproductive potential and emphasizing the association of IUA with infertility [15]. Majority (65.3%) of patients with IUA, significantly had education only up to the secondary school level, which was more than double the number of those with tertiary education. The association of increased incidence of IUA with lower educational status is multifactorial. Lower educational status is more likely to lead to ignorance of appropriate health-seeking behavior or options when these teenagers or women are faced with health challenges. From a prevention or primary health care point of view, contraceptive usage or uptake is likely to be lower in this group, and this may then result in increased incidence of unwanted pregnancies. Taking into account the organization of health services and prevailing laws on induced abortions in Africa and other developing countries, this is likely to lead to procurement of backstreet and unsafe abortions, with resultant infection, trauma in this category of patients, and hence an increased incidence of intrauterine adhesions. Another reason could be that since lower educational status correlates with lower socioeconomic status in most developing countries, economic empowerment and ability to seek the appropriate healthcare in clinical scenarios such as irregular menstruation or infertility would be hindered [1, 4]. Thus such teenagers, adolescents, or women may patronize quacks or alternative medical practitioners who may then inflict harm on them via upper genital tract trauma or unnecessary dilatation and curettage. Even when this group of patients seek health attention from qualified medical practitioners, lower educational status is likely to hinder differentiation between a general practitioner and a qualified specialist gynaecologist. Again, those with lower educational status are less likely to comprehend the reasons for and implications of inadequate dosaging of prescribed antibiotics for pelvic infections, either in number, dose, or duration. Also the higher the educational status, the more likely that the teenagers, adolescents, or women will obtain their antibiotic and other medication from standard outlets such as pharmacies, taking into cognisance the prevalence of substandard and fake medication in this region of the world.

Most of the patients (90.3%) had abnormal menstruation as Ogedengbe et al. found in Lagos. Therefore the index of suspicion for IUA should be high in women of reproductive age with menstrual abnormalities. One patient had normal menstruation and the case was only discovered during the course of investigations for infertility.

 Dilatation and curettage was the most significant predisposing factor identified, being present in about 79% of patients with IUA. This figure is higher than that obtained in Lagos, although the data here also includes uterine evacuation for postpartum haemorrhage. This emphasizes that this procedure should be carried out only by qualified medical practitioners properly trained in this regard. Curettage is advocated to be performed through MVA techniques employing blunt instrument techniques, rather than the traditional dilatation and curettage with sharp instruments [1]. The rationale is less trauma and injury to the endometrium in trained hands, the opposite of which is a crucial factor in the pathogenesis of IUA.

## 9. Conclusion

 Intrauterine adhesions though relatively low in incidence at the gynaecological outpatients' clinic at the NHA, represent a preventable cause of infertility, which constitutes a significant bulk of a gynaecologist's workload in Africa. It is associated with lower educational status and low parity. This may be the case in other developing countries, particularly in Africa.

 Increasing educational targets nationally, poverty alleviation, and nationwide retraining in MVA techniques are likely to reduce the incidence of the condition.

## Figures and Tables

**Table 1:** Age distribution of patients with IUA.

Age	No. of patients	Percentage
20–24	5	6.9
25–29	21	29.2
30–34	37	51.4
35–39	8	11.1
40–44	1	1.4

Total	72	100

**Table 2:** Parity distribution of patients with IUA.

Parity	No. of patients	Percentage
0	43	59.7
1	16	22.2
2	7	9.7
3	3	4.2
4	3	4.2

Total	72	100

**Table 3:** Educational status of 72 cases of IUA.

Educational level attained	No. of patients	Percentage
Primary	2	2.8
Secondary	47	65.3
Tertiary	23	31.9

Total	72	100

**Table 4:** Clinical presentation.

Presentation	No. of patients	Percentage
Secondary amenorrhoea	30	41.7
Hypomenorrhoea	27	37.5
Oligomenorrhoea	8	11.1
Cyclical lower abdominal pain	4	5.5
Recurrent abortions	2	2.8
Normal menstruation	1	1.4

Total	72	100

**Table 5:** Predisposing factors in 72 cases of IUA.

Predisposing factors	Number	Percentage
Dilatation and curettage/uterine		
Evacuation	57	79.2
Myomectomy	6	8.3
Caesarean section	5	6.9
Manual removal of placentae	2	2.8
Pelvic inflammatory disease	1	1.4
Unexplained	1	1.4

Total	72	100
